# Beyond Royalactin and a master inducer explanation of phenotypic plasticity in honey bees

**DOI:** 10.1038/s42003-017-0004-4

**Published:** 2018-01-22

**Authors:** Ryszard Maleszka

**Affiliations:** The Australian National University, Canberra, ACT 2601 Australia

## Abstract

Distinct female castes produced from one genotype are the trademark of a successful evolutionary invention in eusocial insects known as reproductive division of labour. In honey bees, fertile queens develop from larvae fed a complex diet called royal jelly. Recently, one protein in royal jelly, dubbed Royalactin, was deemed to be the exclusive driver of queen bee determination. However, this notion has not been universally accepted. Here I critically evaluate this line of research and argue that the sheer complexity of creating alternate phenotypes from one genotype cannot be reduced to a single dietary component. An acceptable model of environmentally driven caste differentiation should include the facets of dynamic thinking, such as the concepts of attractor states and genetic hierarchical networks.

## Abstract (web summary)

In honeybees, genotypically identical females develop into queens or sterile workers, depending on their diets. In this review, Ryszard Maleszka discusses the controversial role of the royal jelly protein Royalactin in caste determination and provides a framework for moving beyond the master inducer concept.

## Introduction

Many organisms have the capacity to produce contrasting organismal outcomes from one genotype using intricate developmental cues^1–3^. This captivating biological phenomenon known as phenotypic plasticity is found in both plants and animals, particularly in insects, and is considered one of the most interesting albeit poorly understood properties of biological systems. The original concept was used to describe developmental effects on morphological characters^1,4^, but more recently has been broadly applied to all phenotypic responses to environmental change^5,6^. Numerous cellular mechanisms generating confined or systemic responses are required to accomplish plasticity, including gene transcription and translation, epigenomic modifications, metabolic modulation and hormonal regulation^5–12^. The phasing, specificity and pace of plastic responses are essential for their adaptive value. The particular case when variations in environment induce discrete phenotypes is termed polyphenism, which finds its most striking representation in eusocial insects, such as ants and honey bees^2,5,6,13^.

In advanced social honey bees (*Apis mellifera*) one genetic blueprint is used to produce two types of females by utilising nutritional cues from two distinct feeding regimes^7,14,15^. Following an inflexible embryonic development, newly hatched female larvae are multipotent and can develop either into short-lived functionally sterile workers or long-lived fertile queens with both organisms showing distinct phenotypic features, such as different sensory organs, hind legs, body size, ovaries, etc.^16^. Initially, not only the queen-destined larvae, but also worker larvae receive a certain amount of nutritious jelly, although the worker jelly appears to have lower concentration of sugars and some other ingredients compared to the queen food^17^. However, after 3 days of growth only queen-destined individuals will continue to get unrestricted quantities of a special multifactorial food, produced in head glands of nurse bees, known as royal jelly^14,15,18^ or bee milk^19^. In contrast, worker larvae switch to a simpler diet consisting of pollen and sugars, which ensures that they will become functionally sterile helpers. It has been argued that nutritional stress during development to which worker larvae are subjected after 3 days on rich royal jelly-like diet is a critical factor enforcing major reshaping of the worker’s organismal outcome in particular ovarian function and behavioural physiology^20^. Such nutritional stress associated with differential feeding may have been a driver of evolutionary inventions associated with division of labour and insect sociality^20^. It is a striking example of regulatory processes utilising nutritional impact on developmental reprogramming of multipotent cells and how a specialised diet interacting with a single genotype, mediated by epigenomic changes can generate two contrasting organisms^7,21,22^. Currently, this is the most experimentally accessible model in which a defined environmental stimulus, royal jelly, controls post-embryonic development by means of gene regulation via epigenomic modifiers^7,21,23,24^. This highly successful evolutionary invention has been the topic of numerous studies including micro-array and ultra-deep RNA sequencing often combined with genomics and methylomics (epigenomics) that provided initial impetus to unravelling the intricate genetic network driving mechanistic features of phenotypic plasticity in honey bees^25–27^.

## Caste Determination as an Epigenetic Developmental Process

Developmental processes are regulated by a network of interconnected regulatory circuits with multiple genes expressed in a precise spatio-temporal pattern. It is therefore not surprising that a highly publicised 2011 *Nature* paper in which one protein was deemed to be the singular driver of developmental canalisation of the honey bee queens^28^ has met with some reservations^23,29^. The protein in question, fittingly dubbed Royalactin was suggested to act as the master inducer of queen developmental trajectory via the epidermal growth factor receptor (EGFR), to determine the fate of queen or worker. The sole author of that study Kamakura reinforced this notion by evidence obtained in *Drosophila* in which Royalactin appears to induce increased body size and accelerated development by activating EGFR and p70 S6 kinase signalling pathway.

**Fig. 1:**
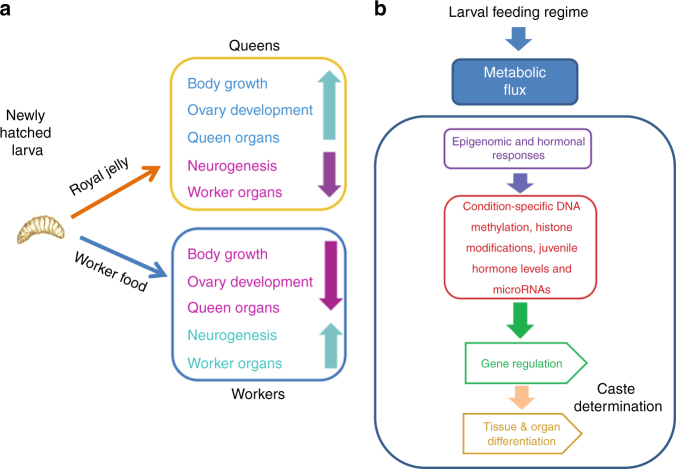
Nutritional programming of postembryonic development in honey bees. **a** Newly hatched female larvae are multipotent and can develop either into short-lived functionally sterile workers or long-lived fertile queens depending on the feeding regime during larval growth. The entire queen development from a fertilised egg to an adult takes 16–17 days. Workers emerge as adults around 5 days later than queens^16^. The distinctiveness in neuronal development in the worker larvae may be associated with the early stages of building a sophisticated nervous system required for workers remarkable navigational skills and high mnemonic fidelity during adult life. **b** Larval feeding regimes act as an external cue that directs epigenetic programing of postembryonic development in a caste-specific manner via metabolic flux. Royal jelly activates pathways associated with the catabolism of proteins, carbohydrates and fats, as well as the major energy pathways. This can be observed as increased growth rates seen in queen larvae relative to that seen in larvae destined to become workers. This process is based on threshold adjustments occurring at several levels, including hormone levels and epigenomic modifications, until a point of no return is reached and development is committed to one phenotype

Royalactin (a monomeric form of Major Royal Jelly Protein 1^30–32^) is one of many components of royal jelly, a unique larval food whose complex composition remains poorly understood^30,33–35^. When a selected female larva is exclusively fed royal jelly it becomes a reproductive long-lived queen. Given the complex nature of this diet and the need for copious amounts of royal jelly over a period of 6 days to produce a mature queen, a conventional explanation of this phenomenon is that a finely tuned feeding regime leads to changes in metabolic flux^26,27^, hormone levels^36–38^ and activation of a cascade of epigenetic regulatory mechanisms including DNA methylation, histone modifications and non-protein coding RNAs, which have the capacity to alter global gene regulation required for producing contrasting organismal outcomes from one genome^7,25–27,39^. Because enzymes responsible for adding or removing epigenetic marks are dependent upon, or influenced by, metabolites, metabolic flux is now recognised as an important driver of DNA and histone modifications and thus a prime mover in gene regulation^24,25,40^ (Fig. 1).

In this context the main findings of Kamakura are quite remarkable because they imply that this epigenetic process is in fact driven by only one ingredient in royal jelly, namely a monomeric form of Royalactin^28^. While the study is impressive in terms of the amount of experimental data, especially using the surrogate *Drosophila* system, it also contains a questionable result on *egfr* methylation^29^ and, conceptually, represents a return to outdated linear molecular simplicity, a point criticized by many authors^22,41–43^. In discussing the implications of Royalactin, the author overlooks the vast trove of data on conditional phenotypes^1–6,14^, the quantitative biological constraints that are manifested during development and the inherent pitfalls in data transferability between different species at particular levels^42^. The evident omission of such relevant datasets in understanding Royalactin’s activities is one reason for confusion surrounding the Royalactin story. Given a huge interest in phenotypic plasticity and genotype–environment interactions, it was only a matter of time before a follow-up study on the proposed role of Royalactin would be available. In a recent article also published in *Nature*, Buttstedt et al.^23^ describe their unsuccessful attempts to repeat some of the original Royalactin experiments and conclude that this protein “is not a royal making of a queen”, effectively suggesting that the 2011 *Nature* results are invalid. In a rebuttal letter accompanying this story, the author of the 2011 study, Kamakura, forcefully argues that the experimental design in Buttstedt et al.^23^ ignores a critical aspect of royal jelly as a determinant of queen fate, namely its quantity^44^. While Kamakura might be right that Buttstedt and colleagues^23^ used smaller quantities of royal jelly in their in vitro experiments than those used in the 2011 *Nature* paper^17^, this explanation does not change the fact that Royalactin cannot induce queen phenotype unless a critical amount of royal jelly is present, suggesting that instead of being a unique control button it acts as one of several important dietary components whose concerted action is required to enforce the queen’s developmental trajectory.

**Fig. 2:**
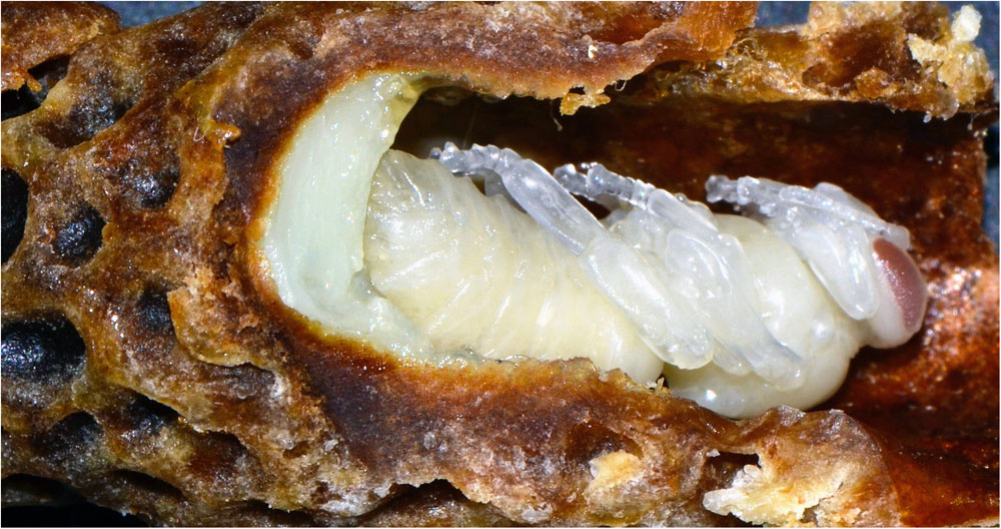
Queen development. A queen pupa undergoing metamorphosis in a special sealed cell that was initially full of royal jelly. The excess of food the queen larva receives is highlighted by some royal jelly left at the bottom. At the pupal stage no food is consumed

To fully appreciate the complexity of this issue it is important to highlight several highly relevant aspects of larval development in honey bees: First, all newly hatched female larvae are initially fed a multifactorial diet (royal jelly or worker jelly^14,15,17^), but after 3 days of growth, only the larva destined to become queen will receive copious amounts of royal jelly^14,45^ (Fig. 2), the quantitative component is critical, as emphasised by Kamakura^44^ in his response to Buttstedt et al.^23^ Indeed, larvae grown in the lab on lower concentrations of royal jelly do not develop all queen features and are characterised as intermediates^7,23,46^. Furthermore, larvae grown on royal jelly deprived of Royalactin also develop queen or queen-like characteristics^23^.

Second, until day 4 of growth, the developmental trajectory that will lead to a queen is reversible^14,45^, suggesting that the initial 3-day exposure of a selected larva to royal jelly is not enough to trigger queen development and that the resulting developmental heterochrony is a gradual threshold-based process driven by instructional vectors from nutritional input rather than by a single on–off switch^7,26^.

Third, a queen can be experimentally induced by interfering with the methylation machinery in newly hatched larvae^47^, and possibly by other means that disturb the developmental network driving phenotypic outcomes. Queen fate is associated with elevated levels of mTOR and RNAi inhibition of this important growth signalling gene induces worker characteristics in queen-destined larvae^48^. Importantly, similarly to the effects of lower quantities of royal jelly, these interventions often generate a gradient of phenotypes referred to as ‘inter-castes’^23,37,46–48^.

Fourth, royal jelly is exceedingly rich in both methionine and methyl groups and some of its major proteins are unusually rich in this essential amino acid, notionally providing substrates for methylation activities^49,50^. However, because of the circular nature of the methionine biosynthetic pathway, methionine excess may actually impair DNA/RNA methylation by inhibiting re-methylation of homocysteine^51^.

Fifth, the fatty acid components of royal jelly are unusual and uncommon structures that are not destroyed at 40 °C, the temperature used by Kamakura to inactivate Royalactin^28^. Some of them exhibit powerful histone deacetylase inhibitory activities^7,52^, which act as chromatin openers affecting the expression of hundreds of genes from the very moment every young larva gets a mouthful of royal jelly. The perceived queen-stimulating properties of small molecules in royal jelly have been considered in a number of studies, always in conjunction with sufficient amounts of food. In one study, an ethanol soluble, protease resistant fraction of royal jelly has been proposed to contain a “queen determinant” on the basis of its capacity to facilitate induction of a queen phenotype in lab experiments^53^.

Sixth, the appropriate control for these experiments to determine the extent to which other proteins and smaller molecules in royal jelly are important for queen development would be to use pure Royalactin in combination with a synthetic diet free of the natural ingredients in royal jelly, admittedly a rather unattainable task.

Seventh, *Drosophila* is indeed facile in terms of experimental manipulation, but it has major drawbacks that impinge upon data transferability (e.g., the lack of DNA methylation toolkit and reproductive division of labour^54^). Phenotypic outcomes depend absolutely on the genetic background used in the experiments^55–57^. As just one clear example reveals, in *mushroom body miniature*, these important brain structures in the fly degenerate in one genetic background, but are completely normal in another^58^. Indeed, it has been shown that royal jelly/Royalactin effects in *Drosophila* are strain-dependent implicating genetic background and cryptic sequence variants as an important factor in phenotypic outcomes driven by the heterologous protein^57^. It is mandatory therefore that if *Drosophila* is to be a surrogate, than the Royalactin experiments need to be conducted in different genetic backgrounds. Notably, royal jelly-based diet is not only atypical, but also toxic for *Drosophila* and even at concentrations much lower than those used for bee larvae has strong detrimental effects on life span, productivity and can negatively affect developmental processes^57,59^. In addition, low levels of royal jelly show similar enhancement for both males and females^59^, whereas a female-specific effect would be expected if it was acting through an analgous pathway as in honey bees.

Finally, to perturb development in *Drosophila*, an organism in which Royalactin does not occur, is akin to perturbing a system with biologically based drugs; one learns about the perturbation, but its biological relevance depends absolutely on the network structure and network flux of the recipient^60,61^. These are quantitative properties of networks, not all or none phenomena (discussed in more detail later on).

In this context, the paper by Grandison et al.^62^ on the effects of amino acid imbalance on longevity and reproduction may have far reaching implications for nutritionally controlled development of queen bees, in particular for caloric restriction, specific metabolic requirements and regulation via mechanisms involving methyl groups. For example, in contrast to many organisms in which dietary restriction promotes longevity but impairs fecundity^63,64^, queen bees are an exception. The rich royal jelly diet of a queen bee makes her one of the most fecund animals on the planet, yet she lives 10–20× longer than her sterile genetically identical workers^49,50,65,66^. The finding by Grandison et al.^62^ that in *Drosophila*, methionine alone is necessary and sufficient to increase fecundity as much as does full feeding, but without reducing lifespan, is striking. As mentioned above, the larval and adult queens’ only food (royal jelly), is very rich in both methionine and methyl groups. In addition to free methionine^49^, some of its major proteins belonging to the Major Royal Jelly Protein (MRJP) family to which Royalactin also belongs are unusually rich in this essential amino acid; MRJP5 contains 68 Met residues (11.5%)^50^, while acetylcholine in royal jelly is six-fold higher than that found in the insect brain^65^. Choline is a hydrolysis product of acetylcholine and is a rich source of methyl groups that could be utilised in regulatory pathways controlling the balance between metabolic and reproductive demands.

Clearly, it would be informative to investigate the effects of high methionine content in royal jelly and relationships between caloric restriction, methylation and acetylcholine with longevity and physiology to better understand how by fine tuning the queen’s diet, honey bees successfully maximised the fecundity of a focal individual in a colony without compromising her life span. Such analyses would undoubtedly advance our efforts to address the unresolved questions regarding the role of Royalactin in phenotypic polymorphism of female honey bees.

Another important aspect to consider in the context of the presumed role of Royalactin in development is the extent to which its in vivo conformation translates to signalling effectiveness in modulating various cellular processes. Royalactin is a monomeric form of MRJP1, an abundant glycoprotein that co-purifies from royal jelly with a small peptide Apisimin, which in turn facilities noncovalent assembly of MRJP1/Apisimin into oligomers^31^. Recent high-resolution structural data have revealed a rather complex picture of this interaction that also involves glycosylation acting as an aggregation inhibitor^32^. The authors conclude that the semi-unfolded structure of MRJP1_Apisimin aggregates may be advantageous for ensuring efficient hydrolysis in the queen larval gut, which notably contains different enzymes than a worker larval intestinal tract^67^.

Intriguingly, the application of the CRISPR technology to produce knock-out mutations in the gene encoding Royalactin yielded viable adult individuals with no obvious phenotypic abnormalities^68^. While this result does not contradict the role of dietary Royalactin in post-embryonic regulatory functions during queen differentiation, it suggests that endogenous Royalactin is dispensable for development. Since this gene also is expressed in the brain^69^ it would be interesting to determine if brain function is affected in mutated individuals.

## Towards an Experimentally Testable Model of Queen Development

Development is particularly vulnerable to opposing trepidations, with multiple downstream outcomes, and this is why phenotypic plasticity is directly linked to development^5^. A certain level of noise in development is expectable and may even have consequences for adaptation^70^. Several authors emphasised the importance of the so-called hierarchical gene regulatory networks (GRNs) and their biological properties, e.g. dynamic stability that is capable of containing excessive stochastic noise^70,71^. Hence, a credible understanding of how phenotypic plasticity evolves should reflect the characteristics of developmental GRNs. A constructive insight into the opportunities here can be gained by taking into account the concept of basins of attraction, a term invented by mathematicians^72^, but often used in the context of gene regulatory networks^61,73^. This idea is best explained by an analogy to a ball bearing moving around the bowl until eventually resting at the lowest point, or point of ‘attraction’. That attractiveness is only effective within a certain space or structure termed the basin or state of attraction for that system, because a ball will move towards a different point if removed from the bowl.

**Fig. 3:**
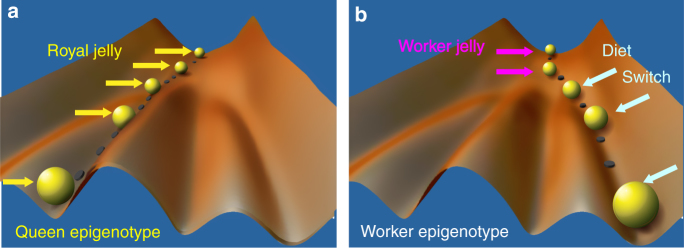
A dynamic view of caste determination inspired by Waddington’s epigenetic landscape. A newly hatched female larva, depicted as a yellow ball, moves along a certain trajectory following instructions from the diet she receives from nurse bees. The valleys represent the basins of attractions, which are the most optimal developmental states under the given set of conditions. A larva fed exclusively royal jelly will become a queen (**a**), whereas a dramatic switch to a less nutritious diet after 4 days will force the larva to take another path resulting in a worker phenotype (**b**). Although both castes are produced from the same genotype, they have different epigenotypes, described by Waddington as a complex of developmental processes that connect genotype and phenotype, or a set of organising principles to which a certain tissue will be subject during development^81,82^

Excessive feeding with royal jelly leads to a major perturbation (noise) of metabolic processes in a larva, which is manifested by an initial slower growth of a queen-destined larva in comparison to worker larvae^74^. The initial noise is rapidly buffered by GRNs and their dynamic stability. As a result, the global network’s topology is remodelled to fit the current instructional vectors from nutrition, which translates into rapid growth and acceleration of queen development. This type of developmental divide is an excellent example of an epigenetic process, whereby external factors generate multiple functional versions of a genome, or epigenomes without affecting the DNA base sequence. One way of visualising the resulting developmental canalisation is by using Waddington’s imaginative allegory of an epigenetic landscape^75^. Waddington’s original ‘epiegenetic’ notions were about the study of the causal developmental mechanisms linking the genotype and the phenotype and understandably were very general as he could not explain how the environmentally modified gene function can generate lasting, “canalised” reactions. However, a modern interpretation of his ideas encompassing the concepts of dynamic thinking and dynamic systems^76^ fits well with the original concept of the epigenetic landscape^77–79^. As shown in Fig. 3, the choice of the two alternate developmental trajectories can be imagined as a growing honey bee female larva (depicted as a yellow ball) travelling across a landscape of mountains and valleys where the valleys represent ‘attractor states or basins’^24,80^. In this illusory landscape, a developing organism travels along an irregular terrain of phenotypic attractors following a set of instructional vectors until it reaches its final state that is said to be optimal under a given set of conditions^79^. As noted by some authors, the epigenetic landscape has some important characteristics that are relevant to epigenotype dynamics: it displays canalisation, demonstrates critical periods when particularly big changes can be induced and shows developmental branching, which lead to clearly distinguished alternative tissues^78^. Indeed, Waddington’s definition of the epigenotype as the set of organising rules or processes linking genotype and phenotype to which various tissues are subjected during development remains fully applicable to modern epigenetics^81,82^. Predictably, the capacity to buffer the developmental trajectory of a female larva can be compromised if there is environmental or dietary change. In laboratory experiments in which various diets were used, the so-called inter-castes with a mixture of queen and worker phenotypic features have been found with significant frequency^23,46,47^. In those cases a new set of instructional vectors shifted the growing larva towards a new attractor state. The striking sensitivity of developmental programming to even small external changes (even in the presence of Royalactin) is yet another argument against a single master regulator. Such developmental de-canalisation/re-canalisation is possible because of a high level of degeneracy in biological systems that provide organisms with the ability to change^22,83^.

Elegant work by the network theory pioneer Barabasi and his colleagues has expanded the protein’s role into “an element in a network of protein–protein interactions, in which it has a contextual or cellular function within functional modules”^84^. They have provided seminal evidence for this idea by showing that the phenotypic consequence of a single gene deletion is affected to a large extent by the topological position of its protein product in the complex hierarchical web of molecular interactions. Both queen and worker trajectories use the same molecular components to achieve plasticity, e.g., insulin signalling, mTOR, juvenile hormone, vitellogenin, etc., that are epigenetically regulated in a context-dependent manner^8–10,26,27,36,85^. One challenge in this field is to find matching molecular and cellular criteria that will allow filtering of the irrelevant network nodes (gene products) or those whose effects on network fluxes are phenotypically minimal, away from those that are bona fide drivers of queen development.

The importance of network modularity for developmental plasticity was recognised by West-Eberhard in her modern interpretation of Waddington’s ideas^86^. By examining the properties of the topology of regulatory networks in queen and worker larvae it should be possible to identify those interconnecting module nodes that are sources of innovation in the evolution of phenotypic dimorphism in honey bees^78^. Such inter-modules may belong to a less-conserved category of network nodes that provide connectivity between partners in different modules in contrast to highly conserved nodes that have tight connections within individual modules^87^. One possibility is that the queen specifying mechanisms evolve relatively quickly and operate via interconnecting modules representing more recent evolutionary novelties. The goal of unravelling the underlying mechanisms is attainable by analysing both gene expression and epigenomic changes using frequent sampling of queen, worker and inter-caste larvae from the moment of hatching up to pupation. Particular attention needs to be given to the clusters of committed, yet undifferentiated progenitors of adult structures in female honey bees called imaginal discs^88^. These pluripotent cells are highly flexible and their fate can be easily manipulated^89^, suggesting that their responsiveness to instructional vectors is frequently being refreshed. Technological innovation is no longer a limiting step in analysing genomic or epigenomic changes^90–93^. When properly analysed, such combined datasets of transcriptomes, methylomes, histone modifications, microRNAs and metabolomes would reveal temporal changes in network topologies relevant to each situation. This kind of analysis, albeit on a smaller scale, based on a microarray transcriptional profiling of queen and worker larvae^27^ has already shown great promise in untangling the differences in caste-specific regulatory networks. Specifically, it has shown that worker’s network is more interconnected than queen’s network suggesting that the worker differentially expressed genes share much more conserved *cis*-elements when compared to queen differentially expressed genes. This result indicates that workers’ genes are more strongly interrelated.

## Conclusion

Crediting a single compound, such as one of the nine highly conserved MRJPs^50^ to be the sole driver of the honey bee queen development is like crediting resveratrol to be the magic ingredient responsible for the so-called “French Paradox” whereby eating a diet high in “bad” fats can be healthy if it is accompanied by red wine^94^. The obvious question is why honey bees would risk a collapse of their social structure by opting for a single protein to control one of the most critical aspects of their life cycle, namely the reproductive division of labour. In addition to being the only reproductive individual in the entire colony, the queen has to ensure that workers’ potential to lay unfertilised eggs is inhibited via a complex mechanism involving pheromones and a highly conserved Notch signalling pathway^95^. In the specific case of its inputs into the mTOR nutrient sensing network^48,96^, Royalactin is simply one of very many components that contribute to network flux. Obviously, it has a defined and important role in this process, but until the points detailed above have been addressed, it is neither special, nor unique.

More research is badly needed to put the role of Royalactin in a proper context and to create a testable model of caste determination in honey bees. To accelerate progress, what is now required is a convergence of advanced molecular techniques with facets of dynamic thinking, attractor states and the concept of an emergent self-organising system. By taking these notions into consideration we should be able to better understand how a continuously refreshed epigenetic landscape provides instructions for developmental decisions to build different body plans. Applying dynamic thinking to honey bee postembryonic development provides a way forward to experimentally advance a research area that is shrouded in an aura of what most likely is an unnecessary controversy.
